# Simultaneous solid–liquid separation and primary purification of clavulanic acid from fermentation broth of *Streptomyces clavuligerus* using salting‐out extraction system

**DOI:** 10.1002/elsc.202000091

**Published:** 2021-07-29

**Authors:** Xu‐Dong Wang, Chun‐Yan Hu, Chao Qin, Yue‐Sheng Dong, Guo‐Qing Ying, Zhi‐Long Xiu, Zhi‐Guo Su

**Affiliations:** ^1^ School of Bioengineering Dalian University of Technology Dalian P. R. China; ^2^ College of Pharmaceutical Science Zhejiang University of Technology Hangzhou P. R. China; ^3^ State Key Laboratory of Biochemical Engineering Institute of Process Engineering Chinese Academy of Sciences Beijing P. R. China

**Keywords:** clavulanic acid, fermentation broth, salting‐out extraction, solid–liquid separation

## Abstract

Clavulanic acid (CA) is usually used together with other β‐lactam antibiotics as combination drugs to inhibit bacterial β‐lactamases, which is mainly produced from the fermentation of microorganism such as *Streptomyces clavuligerus*. Recently, it is still a challenge for downstream processing of low concentration and unstable CA from fermentation broth with high solid content, high viscosity, and small cell size. In this study, an integrated process was developed for simultaneous solid–liquid separation and primary purification of CA from real fermentation broth of *S. clavuligerus* using salting‐out extraction system (SOES). First, different SOESs were investigated, and a suitable SOES composed of ethanol/phosphate was chosen and further optimized using the pretreated fermentation broth. Then, the optimal system composed of 20% ethanol/15% K_2_HPO_4_ and 10% KH_2_PO_4_ w/w was used to direct separation of CA from untreated fermentation broth. The result showed that the partition coefficient (*K*) and recovery yield (*Y*) of CA from untreated fermentation broth were 29.13 and 96.8%, respectively. Simultaneously, the removal rates of the cells and proteins were 99.8% and 63.3%, respectively. Compared with the traditional method of membrane filtration or liquid–liquid extraction system, this developed SOES showed the advantages of simple operation, shorter operation time, lower process cost and higher recovery yield of CA. These results demonstrated that the developed SOES could be used as an attractive alternative for the downstream processing of CA from real fermentation broth.

**Abbreviations**
ATPMS: aqueous two‐phase mixed micellar system‐
ATPS: aqueous two‐phase system‐
CA: clavulanic acid
SOE: salting‐out extraction
SOES: salting‐out extraction system

## 1 INTRODUCTION

The bacterial infection is a global healthy problem. One of the successful methods to overcome this problem is to use β‐lactamases inhibitors such as clavulanic acid (CA) [1, 2]. CA is clinically effective and functions a substrate analogue which covalently binds to the active centers of the bacterial β‐lactamases, thus inactivating them irreversibly. Currently, CA is mainly used together with other β‐lactam antibiotics such as amoxicillin to inhibit bacterial β‐lactamases in pharmaceutical industry [3].

Industrially, CA is mainly obtained by the fermentation of microorganism such as *Streptomyces clavuligerus* [2, 4]. The downstream processing of CA usually requires three processes including solid–liquid separation, primary purification and fine purification. Due to the high solid content, high viscosity, small cell size, and low concentration of unstable CA of fermentation broth, the solid–liquid separation and primary separation process remains a great challenge.

For solid–liquid separation of CA, traditional methods such as high‐speed centrifugation or plate filtration are not suitable to clarify such complex fermentation broth due to their low efficiency, which require additional pretreatment steps such as flocculation and precipitation. In industry, solid–liquid separation of CA from fermentation broth is recently implemented by three membrane filtration: microfiltration to remove all of the cells, ultrafiltration to remove most of proteins, and nanofiltration to concentrate the CA [2]. However, new CA degradation impurities are produced in these process operations, due to long operation time and instability of CA [5, 6, 7, 8]. Besides, the membrane fouling is easy to occur because of the above mentioned properties of *S. clavuligerus* fermentation broth. Therefore, the development of a fast and efficient method for solid–liquid separation of CA from fermentation broth is still urgently needed.

For primary purification of CA from the clarified fermentation broth after solid–liquid separation, water‐immiscible organic solvent based liquid–liquid extraction [2] or adsorption [9] has been used. However, this process has a drawback of low purification yield of CA due to the property of absence of strongly hydrophobic groups and instability of CA. To improve the purification efficiency, polymer (e.g., PEG)/salt or polymer/polymer based aqueous two‐phase systems (ATPSs) [10, 11, 12, 13] or aqueous two‐phase mixed micellar systems (ATPMSs) [14, 15, 16] have been used to purify CA from fermentation broth in recent years. However, the polymer has relatively high viscosity and cost, which leads to this ATPS being rarely used on an industrial scale. As an alternative, hydrophilic organic solvent (e.g., a short‐chain alcohol)/salt based SOES, which has the advantages such as low solvent viscosity and cost, easy solvent recovery, fast phase separation rate and easy scale up, has been proven as an efficient method for primary purification of low concentration and high hydrophilicity molecules from complex substrates [17, 18, 19, 20, 21, 22, 23, 24, 25, 26, 27, 28, 29, 30, 31]. Therefore, this SOES approach is a worth trying to separate the CA from *S. clavuligerus* fermentation broth because of the similar features of these complex substrates.

On the other hand, process integration, which emphasizes the combination of two or more unit operations by one single‐unit operation to reduce operating steps and costs [32], is also an alternative. Interestingly enough, this SOES technology has been efficiently used to integrate the solid–liquid separation and primary purification of some target biomolecules from complex fermentation broth into a single unit operation [17, 28, 31].

Therefore, the aim of this study was to develop a suitable SOES to integrate the solid–liquid separation and primary purification of CA from *S. clavuligerus* fermentation broth, thus improving the process efficiency (e.g., shortened operation time, improved purification efficiency and recovery rate of CA). First, different SOESs were screened using pretreated fermentation broth and the most suitable one was further optimized. Secondly, simultaneous solid–liquid separation and primary purification of CA from untreated fermentation broth was investigated using the chosen SOES. Finally, the process efficiency of the developed SOES strategy was compared with the traditional methods of membrane filtration or liquid–liquid extraction system.

## 2 MATERIALS AND METHODS

### 2.1 Materials

Fermentation broths were provided by Sinopharm Weiqida pharmaceutical Co., Ltd. (Datong, China). There were two different sources of *S. clavuligerus* fermentation broths containing CA: (1) One was untreated fermentation broth containing the cells and proteins, which had a CA concentration of 7 g/L. (2) The other was pretreated fermentation broth (filtrate) through microfiltration, ultrafiltration and nanofiltration to remove all of the cells and most of proteins, which had a CA concentration of 15 g/L. CA standard in its potassium salt form was also supplied by Sinopharm Weiqida pharmaceutical Co., Ltd. (Datong, China). Ethanol and other reagents were purchased from Sinopharm Chemical Reagent Co., Ltd. All other reagents are analytical grade.

### 2.2 Salting‐out extraction of CA from pretreated fermentation broth (filtrate)

Each extraction experiment at a total mass of 20 g was carried out in 25‐ml graduated centrifuge tubes by addition of appropriate amounts of pretreated fermentation broth (filtrate), salts, and organic solvents. This mixture solution was vortex‐mixed for 5–10 min, and then was statically placed until complete phase separation at room temperature. After phase separation, the phase volumes were noted and the concentrations of CA in organic solvent‐rich (top) and salt‐rich (bottom) phases were determined by a RP‐HPLC method as described in later Section 2.4.

SOESs composed of four organic solvents including ethanol, propanol, n‐butanol and ethyl acetate and two buffer salts including K_2_HPO_4_ and KH_2_PO_4_, citric acid and sodium citrate were investigated.

The phase ratio (*R*) was defined as the ratio of the volume of the top phase to that of the bottom phase. The partition coefficient (*K*) was defined as the ratio of the concentration of CA in the top phase to that in the bottom phase. The recovery yield (*Y*, %) was defined as the mass ratio of CA partitioned in the top phase to the total amount of CA. The mass balance (*MB*, %) was defined as the ratio of the total mass of CA in top and bottom phase to the initial total mass of CA. These parameters were calculated as follows:

(1) $$R=\frac{V_{t}}{V_{b}}$$

(2) $$K=\frac{C_{t}}{C_{b}}$$

(3) $$Y\left(\%\right)=\frac{C_{t}\times V_{t}}{M}\times 100$$

(4) $$MB\left(\%\right)=\frac{C_{t}\times V_{t}+C_{b}\times V_{b}}{M}\times 100$$

where *V_t_* and *V_b_* are the volumes of the top and bottom phases, *C_t_* and *C_b_* are the concentrations of CA in the top and bottom phases, and *M* is the original quantity of CA in the fermentation broth.

**PRACTICAL APPLICATION**

This study demonstrated an efficient process for simultaneous solid–liquid separation and primary purification of clavulanic acid (CA) from real fermentation broth of *S. clavuligerus* using an ethanol/phosphate based salting‐out extraction system (SOES). This process was accomplished within 1 h, achieved a CA recovery yield of 96.8% as well as removal rates of the cells and proteins of 99.8% and 63.3%, respectively. The developed SOES technology has the advantages of faster phase separation rate, lower solvent viscosity and cost, and easier solvent recovery, compared with the traditional liquid–liquid extraction system such as PEG/salt based aqueous two‐phase system. This technology also has the advantages of simpler operation, less process cost and easier scale up, compared with the traditional membrane filtration. These results demonstrated the developed SOES technology as a promising tool for the direct recovery of CA from untreated fermentation broth on an industrial scale.

### 2.3 Salting‐out extraction of CA from untreated fermentation broth

SOE of CA from the untreated fermentation broths followed the procedures as described above for the pretreated fermentation broth (filtrate) with a minor modification. That is, low‐speed centrifugation (3000 × *g* for 10 min) was used to facilitate the phase separation. The samples were collected and analyzed for the determination of CA in top and bottom phases, and the removal rates of cells and proteins were also determined.

### 2.4 Analysis method

CA concentration was determined by RP‐HPLC method through measuring the release of the reaction product between CA and imidazole, (1‐[8‐hydroxy‐6‐oxo‐4‐azooct‐2‐enol]‐imidazole) [33]. The RP‐HPLC analysis was performed on a SinoChrom ODS‐BP (5 μm, 4.6 mm × 200 mm) using an Agilent 1100 HPLC system (Agilent Technology, CA, USA) at detection wavelength of 312 nm, flow rate of 1.0 ml/min, sample volume of 10 μl and column temperature of 25°C. The regression equation was *Y *= 8563.84*X* + 87.76 (*R* ^2 ^= 0.9997) at the range of 0.10–1.00 g/L, where *X* was the concentration of CA (g/L) and *Y* was the peak area.

Protein concentrations in the fermentation broth and organic phase from SOES were determined by the Kjeldahl method using a VELP UDK159 type automatic azotometer [34].

The biomass concentration was measured by monitoring the absorbance at 600 nm using a spectrophotometer [35].

## 3 RESULTS AND DISCUSSION

### 3.1 Screening of SOES using pretreated fermentation broth

In the industrial purification process of CA, membrane filtration was used for the pretreatment of the broth to remove bacteria and proteins, and then successive liquid–liquid extraction steps (generally employing an organic solvent like ethyl acetate or methyl isobutyl ketone) were conducted at pH 1.5‐2.0 (CA pKa = 2.3–2.7 [36]). This process not only produced a large amount of acid waste water, but also caused severely degradation of CA, because of instability of CA at this pH range [5, 6]. Therefore, the partition behaviors of CA in different SOESs composed of two kinds of buffer salts (K_2_HPO_4_/KH_2_PO_4_, and citric acid/sodium citrate) and four organic solvents (ethanol, propanol, n‐butyl alcohol, and ethyl acetate) were investigated, adjusting the pH in the range of 6–8, in which CA is relatively stable. As shown in Table 1, the partition behaviors of CA in different SOESs varied greatly. The partition coefficient (*K*) and recovery yield (*Y*) of CA increased with the hydrophilicity of the organic solvent. This result was similar to a reported study [37], in which the solubility of CA in its potassium salt form ranked in the following order: ethanol > 1‐propanol > 1‐butanol > 2‐propanol > 2‐methyl‐1‐propanol. Since CA exists in the dissociation form when the system pH > 2.3 [5, 6], hydrophobic organic solvents such as ethyl acetate had the difficulty in the extracting of CA into the top phase at these screened systems with pH range of 6–8 (Table 1). Compared with citrate based system, phosphate based system exhibited a better extraction efficiency of CA. Among these SOESs, ethanol/phosphate based SOES had the best extraction efficiency of CA, thus being chosen for subsequent study.

**TABLE 1:** Partition behaviors of CA in the SOESs composed of different salts and organic solvents

SOES	Salt/organic solvent (%, w/w)	*R*	*K*	*Y* (%)
Phosphate^a^/ethanol	10/25	9.83 ± 0.12	127.07 ± 3.18	82.6 ± 1.9
	15/25	4.31 ± 0.08	40.80 ± 1.64	77.9 ± 2.2
	20/25	1.57 ± 0.05	35.74 ± 2.05	44.8 ± 1.5
Phosphate/n‐propanol	10/25	0.64 ± 0.03	1.03 ± 0.06	23.9 ± 0.8
	15/25	0.77 ± 0.04	1.34 ± 0.05	28.8 ± 1.1
	20/25	0.80 ± 0.02	1.94 ± 0.08	33.3 ± 1.6
Phosphate/n‐butanol	10/25	0.66 ± 0.05	0.06 ± 0.01	2.6 ± 0.3
	15/25	0.64 ± 0.03	0.06 ± 0.01	2.1 ± 0.4
	20/25	0.75 ± 0.04	0.08 ± 0.01	2.8 ± 0.2
Phosphate/ethyl acetate	10/25	0.43 ± 0.01	0^c^	0
	15/25	0.50 ± 0.02	0	0
	20/25	0.57 ± 0.04	0	0
Citrate^b^/ethanol	10/25	‐^d^	–	–
	15/25	4.49 ± 0.16	1.53 ± 0.07	72.0 ± 1.5
	20/25	1.33 ± 0.10	3.17 ± 1.12	68.3 ± 2.3
Citrate/n‐propanol	10/25	0.47 ± 0.03	0.98 ± 0.04	20.7 ± 0.7
	15/25	0.58 ± 0.02	1.00 ± 0.05	23.7 ± 1.2
	20/25	0.62 ± 0.02	0.99 ± 0.03	25.4 ± 0.8
Citrate/n‐butanol	10/25	0.68 ± 0.04	0.06 ± 0.01	3.4 ± 0.6
	15/25	0.80 ± 0.02	0.11 ± 0.02	6.7 ± 0.4
	20/25	1.10 ± 0.06	0.04 ± 0.00	3.4 ± 0.5
Citrate/ethyl acetate	10/25	0.45 ± 0.02	0	0
	15/25	0.51 ± 0.04	0	0
	20/25	0.59 ± 0.03	0	0

^a^ Phosphate was a mixture of K_2_HPO_4_ and KH_2_PO_4_ (1:1, w/w).

^b^ Citrate was a mixture of citric acid and sodium citrate (1:1, w/w).

^c^ ‘0‘ represented that the CA was not detected in the organic solvent‐rich top phase.

^d^ ‘‐‘ represented that the two‐phase system was not formed.

### 3.2 Partition behavior of CA in an ethanol/phosphate based SOES using pretreated fermentation broth

Because the phase compositions have significant effects on partition behavior of target molecules in SOE [17, 18, 19, 20, 21, 22, 23, 24, 25, 26, 27, 28, 29, 30], the influences of phosphate and ethanol concentrations on the partitioning behaviors of CA were investigated. Moreover, the effects of pH, by changing the proportion of K_2_HPO_4_ and KH_2_PO_4_, on the partition and degradation behaviors of CA were also investigated. As shown in Table 2, the phase ratio (*R*) decreased from 2.11 to 0.73 with increasing salt content at a given ethanol concentration. Additionally, the increase in the amount of K_2_HPO_4_ led to degradation of CA due to the increasing pH, which presented a similar trend to a reported study [6]. The salts composed of 15% K_2_HPO_4_/10% KH_2_PO_4_ were chosen as optimized condition due to the relatively proper phase ratio (*R*) and the highest recovery yield (*Y*) of CA.

**TABLE 2:** Effect of phosphate concentration and composition on the partition behavior of CA in ethanol/phosphate based SOES

Phosphate/ethanol (%, w/w)	K_2_HPO_4_/KH_2_PO_4_ (%, w/w)	pH of organic phase	*R*	*K*	*Y* (%)	*MB* (%)
25/20	12.5/12.5	6.95	2.11 ± 0.11	23.72 ± 1.28	96.4 ± 2.4	97.5 ± 2.3
	15.0/10.0	7.16	1.23 ± 0.08	22.05 ± 0.96	95.9 ± 1.8	96.7 ± 1.7
	17.5/7.5	7.66	1.16 ± 0.05	21.01 ± 1.51	81.5 ± 2.5	84.5 ± 2.4
	20.0/5.0	8.01	1.10 ± 0.03	19.93 ± 1.16	73.7 ± 1.6	76.6 ± 1.9
30/20	17.5/12.5	7.43	1.35 ± 0.04	29.14 ± 0.85	75.7 ± 2.3	77.6 ± 1.8
	20.0/10.0	7.71	0.91 ± 0.01	17.02 ± 1.59	76.4 ± 1.8	81.4 ± 2.0
	22.5/7.5	8.01	0.91 ± 0.03	23.86 ± 1.34	64.1 ± 0.9	67.1 ± 1.3
	25.0/5.0	8.35	0.83 ± 0.05	26.52 ± 1.77	60.7 ± 1.2	63.4 ± 1.6
35/20	22.5/12.5	7.72	0.81 ± 0.08	37.32 ± 2.02	73.9 ± 2.1	76.3 ± 1.5
	25.0/10.0	7.95	0.76 ± 0.04	43.99 ± 1.83	71.2 ± 1.7	73.3 ± 2.1
	27.5/7.5	8.20	0.70 ± 0.07	58.56 ± 0.85	64.4 ± 1.3	66.0 ± 0.9
	30.0/5.0	8.54	0.73 ± 0.02	57.76 ± 1.46	64.8 ± 1.5	66.3 ± 1.3

**FIGURE 1:**
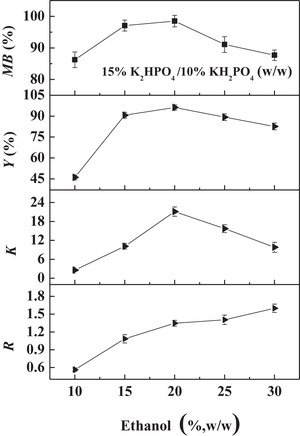
Effects of ethanol concentration on the partition behavior of CA in the ethanol/phosphate based SOES

Then, the effect of ethanol concentration on the partitioning behavior of CA was investigated (Figure 1). The partition coefficient (*K*), recovery yield (*Y*) and mass balance (*MB*) first increased and then decreased with increasing ethanol concentration at a given phosphate concentration. This phenomenon was probably caused by the distribution and degradation behaviors of CA in this SOES [6, 7, 8]. When the ethanol concentration was lower than 20% (w/w), the distribution of CA in the organic phase increased with increasing ethanol concentration, while the degradation of CA decreased due to higher stability of CA in the ethanol–enriched top phase than the salt–enriched bottom phase. When the ethanol concentration was higher than 20% (w/w), more water molecules entered into the ethanol–enriched top phase with increasing ethanol concentration, which probably increased the pH of this SOES due to the increased precipitation of KH_2_PO_4_. Similar trend was observed when the amount of K_2_HPO_4_ increased in Table 2. Therefore, the ethanol concentration of 20% w/w was chosen as optimized condition due to the relatively proper phase ratio (*R*) and the highest recovery yield (*Y*) of CA.

Based on the above results, the optimal condition for extraction of CA from pretreated fermentation broth (filtrate) was 20% ethanol/15% K_2_HPO_4_/10% KH_2_PO_4_ w/w. Under this condition, the partition coefficient (*K*) and recovery yield (*Y*) of CA were 21.11 and 96.2%, respectively (Figure 1).

### 3.3 Salting‐out extraction of CA from untreated fermentation broth

**FIGURE 2:**
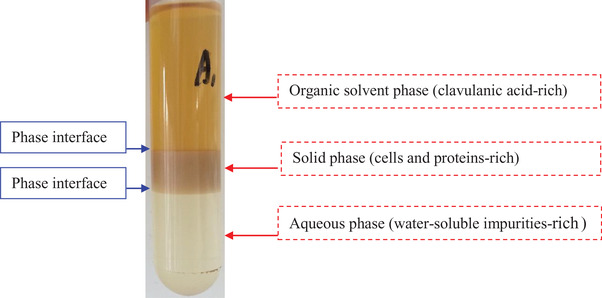
Simultaneous solid–liquid separation and primary purification of CA from untreated fermentation broth of *Streptomyces clavuligerus* using ethanol/phosphate based SOES

**FIGURE 3:**
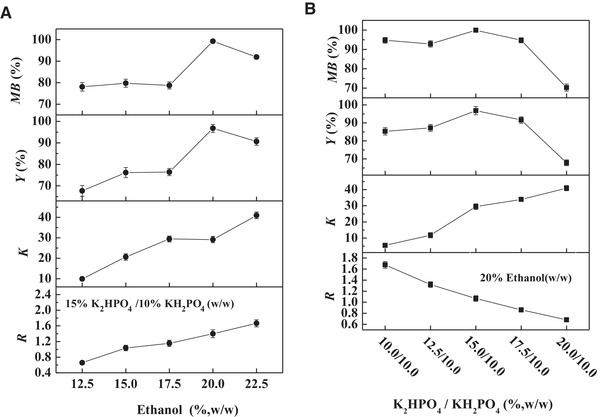
Effects of concentrations of ethanol and phosphate on the separation of CA from fermentation broth using ethanol/phosphate based SOES

To evaluate the feasibility of simultaneous solid–liquid separation and primary purification of CA from untreated fermentation broth of *S. clavuligerus* using the SOES, this process was first carried out at the optimal condition obtained using pretreated fermentation broth. As shown in Figure 2, a three‐phase system, composed of ethanol‐rich top phase, solid‐rich middle phase and phosphate‐rich bottom phase, was formed. Most of CA was enriched in the top phase, whereas most of cells and proteins were enriched in the middle phase. Compared with pretreated fermentation broth, the untreated fermentation broth contains cells and proteins, which might affect the partition behavior of CA, thus the SOES was re‐optimized (Figure 3). The optimized phase composition of 20% ethanol/15% K_2_HPO_4_ and 10% KH_2_PO_4_ w/w was still consistent with that of pretreated fermentation broth (Figure 1). As shown in Figure 3, the partition coefficient (*K*), and recovery yield (*Y*) of CA under this optimized condition were 29.13‐ and 96.8%, respectively. Simultaneously, 99.8% of cells and 63.3% of proteins were removed from the fermentation broth. This property that SOE could remove all of cells and most of proteins from the fermentation broth has also been proven in our previous studies [17, 28, 31, 38]. The above results demonstrated an efficient method for the recovery of CA from untreated fermentation broth, which integrated the solid–liquid separation and primary purification processes into a single unit operation, thus reducing the operation steps and cost.

### 3.4 Comparison of salting‐out extraction and other liquid–liquid extraction systems for the recovery of CA from fermentation broth

Industrially, the clarified fermentation broth by membrane filtration was usually primarily purified using water‐immiscible organic solvent (e.g., ethyl acetate) based liquid–liquid extraction [2]. In this process, the pH of the filtrate should be adjusted to pH 1.5–2.0, which produced a large amount of acid waste water, thus causing the possible environmental pollution. Moreover, this process required a large amount of organic solvent and multistage extraction due to the low extraction efficiency for the lack of strong hydrophobic groups in the CA. To improve the extraction efficiency of CA, more environmental friendly and effective liquid–liquid extraction systems such as aqueous two‐phase systems (ATPSs) [10, 11, 12, 13] or aqueous two‐phase mixed micellar systems (ATPMSs) [14, 15, 16] were developed in recent years, which were summarized in Table 3. Among these systems, the ATPMSs had relatively low distribution coefficients (*K*) and extraction yields of CA, although it had the low solvent consumption. The ATPSs composed of PEG/inorganic salt (e.g., phosphate or citrate), had relatively high distribution coefficients (K) and extraction yields of CA. Relatively, the PEG/sodium polyacrylate (NaPA) based ATPS, even applying [Ch]Cl as adjuvant, had lower extraction yields of CA than that of PEG/inorganic salt based system. However, the PEG based ATPSs were limited on an industrial scale, especially using untreated fermentation broth, because of its relatively high viscosity and cost and the difficulty in the recycling. Compared with the reported systems in Table 3, the ethanol/phosphate based SOES showed a relatively high distribution coefficient (*K*) and extraction yield (*Y*) of CA, even using the untreated fermentation broth. Moreover, this system also has the advantages of faster phase separation rate, lower solvent viscosity and cost, easier solvent recovery.

**TABLE 3:** Comparison of different liquid–liquid extraction systems for the separation of CA

CA source	System composition	*Y* (%)	*K*	*MB* (%)	Ref.
Pretreated fermentation broth	PEG/phosphate	94.0	11.10	−	[13]
Pretreated fermentation broth	PEG/NaPA	55.0	9.15	80.0	[11]
Pretreated fermentation broth	PEG/NaPA+ [Ch]Cl	85.5	5.6	−	[12]
Pretreated fermentation broth	AOT/TX‐114	86.3	1.48	>90	[16]
Simulated solution containing 99% purity CA	PEG/citrate	103.5	5.92	−	[10]
Simulated solution containing 99% purity CA	TX‐100/DX‐S	49.0	1.33	84.0	[14]
Simulated solution containing 54% purity CA	AOT/C_10_E_4_	43.0	−	83.0	[15]
Simulated solution containing 54% purity CA	CTAB/C_10_E_4_	21.5	−	−	[15]
Pretreated fermentation broth	Ethanol/phosphate	96.2	21.11	98.8	This work
Untreated fermentation broth	Ethanol/phosphate	96.8	29.13	98.6	This work

### 3.5 Comparison of salting‐out extraction and membrane filtration for the recovery of CA from fermentation broth

CA has a low chemical stability, which produces new CA degradation impurities over time in fermentation broth containing a large number of impurities [5, 6, 7, 8]. Therefore, the solid–liquid separation and primary purification processes should be accomplished as fast as possible. Industrially, this process has been achieved by three membrane filtration of microfiltration, ultrafiltration and nanofiltration [2]. Unfortunately, the *S. clavuligerus* fermentation broth has relatively high viscosity and small cell size, which should be diluted with a large amount of water to reduce the fouling of microfiltration membrane, thus increasing the processing capacity. After microfiltration and ultrafiltration operations, the filtrate containing CA should be re‐concentrated by nanofiltration membrane. Therefore, this process is time‐consuming. As shown in Table 4, SOE process developed in this study has obvious advantages compared with the traditional membrane filtration process: (1) The operation time was reduced from more than 10 to 1 h. (2) The recovery rate of CA increased from 94.1% to 96.8%. (3) The protein removal rate was also higher, which was better for subsequent fine purification process to obtain the CA of desired purity. Furthermore, SOE technology has a simpler operation, less process cost and easier scale up compared with membrane filtration.

**TABLE 4:** Comparison of SOE and membrane filtration for the separation of CA from fermentation broth

Method	Removal rate of cells (%)	Removal rate of protein (%)	Processing time (h)	*Y* (%)	*MB* (%)
SOE	99.8	64.7	1.0	96.8	99.2
Membrane filtration^a^	99.5	38.7	≥10.0	94.1	98.0

^a^ The data were provided by the Sinopham Weiqida Pharmaceutical Co., Ltd.

Based on the above results, the developed SOE technology in this study might be used as a promising tool for direct recovery of CA from untreated fermentation broth on an industrial scale. Future work will be focused on the recovery and recycling of the phase compositions (e.g., ethanol and phosphate) in the top and bottom phases, which is helpful to develop a more economical and efficient SOES for a real industrial production of CA from fermentation broth.

## 4 CONCLUDING REMARKS

In this study, an integrated process for simultaneous solid–liquid separation and primary purification of CA from fermentation broth of *S. clavuligerus* was successfully developed using the SOES. Using pretreated fermentation broth, several SOESs composed of different organic solvents and salts were screened by evaluating their extraction efficiencies. A suitable SOES composed of ethanol/phosphate was chosen and further optimized. Using untreated fermentation broth, a high partition coefficient (*K*) of 29.13, and high recovery yield (*Y*) of 96.8% for the recovery of CA were achieved under the optimal condition composed of 20% ethanol/15% K_2_HPO_4_ and 10% KH_2_PO_4_ w/w. Simultaneously, 99.8% of the cells and 63.3% of proteins were removed. This developed SOES showed the advantages of simple operation, short operation time, low process cost and high recovery yield of CA, compared with the traditional membrane filtration or liquid–liquid extraction system. This study demonstrated the ethanol/phosphate based SOES as an economic and effective technology for the recovery of CA from untreated fermentation broth with the properties of low concentration of unstable CA, high solid content, high viscosity, and small cell size.

## CONFLICT OF INTEREST

The authors have declared no conflict of interest.

## Data Availability

The data that support the findings of this study are available from the corresponding author upon reasonable request.
